# Validation of a Clinical Instrument for Measuring the Severity of Acute Bronchitis in Children – The BSS-ped

**DOI:** 10.2174/1874306401812010050

**Published:** 2018-10-26

**Authors:** Siegfried Lehrl, Peter Kardos, Heinrich Matthys, Wolfgang Kamin

**Affiliations:** 1 Department of Psychiatry and Psychotherapy, Friedrich-Alexander University Erlangen-Nuremberg, Erlangen, Germany; 2 Group Practice and Centre for Pneumology, Center for Respiratory, Allergy and Sleep Medicine at Red Cross Maingau Hospital, Frankfurt am Main, Germany; 3 Department of Pneumology, University Hospital Freiburg, Freiburg, Germany; 4 Clinic for Paediatrics, Evangelic Hospital Hamm, Hamm, Germany

**Keywords:** Acute bronchitis, Bronchitis severity scale, Clinical psychometry, Outcome assessment, Severity of illness index, Validation

## Abstract

**Background:**

There are no validated standardised clinical procedures for severity measurement of acute bronchitis in children. The “BSS-ped”, a short version of the physician-rated assessment scale BSS (Bronchitis Severity Scale), can fill this gap, if it is valid.

**Objective:**

To examine the scale´s validity.

**Methods:**

Investigations were planned according to classical clinical-psychometric validity criteria including a formal competence evaluation of the scale´s authors and statistical analyses of data from 78 patients aged 1-6 and diagnosed with “acute bronchitis”. Cross-validation was provided by analysis of data from 70 children with matching age, sex and diagnosis. All children were examined three times (day 0, 3-5 and 7) using the BSS-ped in addition to other clinical and psychometric monitoring procedures.

**Results:**

The evidently high level of expertise of the scale’s authors substantiates pronounced content validity and relevance of the BSS-ped and its items. The validity criterion, *i.e*. to reflect the unidimensional severity of acute bronchitis and its change using the BSS-ped score, was fulfilled. There were substantial correlations with other scales measuring the current health-related quality of life, as well as satisfaction and success of treatment. Severity change prognoses for acute bronchitis under placebo and an active substance were correct. The BSS-ped was found to be a feasible instrument because it can be repeated at short intervals (minute range) without any special technical aids or extended training.

**Conclusion:**

The BSS-ped is a valid procedure for measuring the severity of acute bronchitis in children.

## 1 BACKGROUND AND OBJECTIVE

Since 1972, a number of clinical procedures have been published for the standard measurement of the severity of common respiratory disorders in children, *e.g*. bronchiolitis, some of which are frequently used in practice [1-7]. Since 2013, the clinical quality of these measurement procedures has also gained interest in the respective publications [6-9], validity (or validness) in particular. Validity involves proving that a measurement procedure does actually measure what it is supposed to measure, *e.g*. the severity of bronchiolitis in children.

In the field of Acute Bronchitis (AB), which occurs more frequently in comparison to bronchiolitis in pediatric practice [10], the status of the measurement of severity is less favourable. As far as we can judge, there are no assessment scales available apart from the BSS-ped (Bronchitis Severity Scale short version; details in next section). The BSS-ped was applied during the investigation of the efficacy of a pharmaceutical preparation and not examined in terms of its measurement quality characteristics [11].

The BSS-ped is a short version of the Bronchitis Severity Scale (BSS), an observer-rated assessment scale for all age groups that records five major symptoms of acute bronchitis. The symptoms and severity to be assessed by the BSS were already presented in 1996 in two publications by Haidvogl *et al*. [12] and Dome & Schuster [13], although the term “BSS” was not used at that time. This appeared in the literature for the first time in 2003 [14] and was used after that in many other publications.

The following three items, which can be easily assessed by the medical investigator in cooperation with the parents, were selected from the BSS for the BSS-ped: Coughing, pulmonary rales at auscultation, and dyspnoea. Their presence is to be assessed in each case according to a 5-point scale: 0 = absent, 1 = mild, 2 = moderate, 3 = severe, 4 = very severe. The points are summed up to form a total score that can amount to between 0 and 12 points and should indicate the overall severity of AB. This short version of the Bronchitis Severity Scale may be used for pre-school children, in particular.

The objective of the current investigation was to examine whether the most important quality characteristics of the measurement procedures can satisfactorily demonstrate the validity of the BSS-ped.

## 2 PATIENTS AND METHODS

### 2.1 Samples

In an earlier study (randomised, double-blind, placebo *versus* Pelargonium sidoides preparation EPs 7630^1^ as the active substance) [11], the BSS-ped was already used by a total of eleven physicians at eleven study centres in 78 male or female children aged between one to six years with the diagnosis of “acute bronchitis”. The BSS-ped was measured three times for each subject (visit 1 (day 0), visit 2 (day 3-5), visit 3 (day 7)), in addition to other clinical survey procedures [11]. Although this “main sample” served other purposes than the investigation of the quality characteristics of the measurement instrument, the data were available for a post-analysis of this sort.

Moreover, for another randomized, double-blind and placebo-controlled (comparative sample) clinical trial [15], additional data assessed by means of the full BSS could be obtained from the sponsor. This trial was performed in further ten centres and subsequently with ten principal investigators possessing long-term expertise in the field of pediatrics. In this trial, the total BSS, which also contains the items of the BSS-ped, was recorded for 70 male or female children aged one to six years who were also suffering from AB. Here too, the BSS was recorded at visit 1 (day 0), visit 2 (day 3-5) and visit 3 (day 7). The data from this comparative study serve cross-validation for testing the internal structure of the BSS-ped, in order to evaluate whether this measuring instrument actually records the severity of AB.

### 2.2 Validity Criteria

The quality criterion of validity is met if the measurement instrument, *i.e*. the BSS-ped, measures to a large extent what it is supposed to measure. In this case the severity of AB in children who can only be expected to provide little or no information about their condition, depending on their age. Validity testing was to be carried out according to all the important aspects of classical measurement and test theory [16, 17]. These encompass content validity, construct validity and criterion validity.

EPs® 7630 is the active ingredient of the product Umckaloabo® (ISO Arzneimittel, Ettlingen, Germany).

#### 2.2.1 Content Validity and Testing Options

Content validity exists if the content of the items of a measurement instrument defines the target characteristic – here the severity of AB – with sufficient accuracy. Generally, content validity cannot be determined numerically. Rather, it is accepted or rejected on the basis of logical professional considerations [16, 17]. One option that may be used for indirectly testing professional acceptability is to investigate whether the authors of the scale are recognized in the international scientific community. This is apparent from citations of their scientific work by other scientists [18].

#### 2.2.2 Construct Validity and Testing Options

Of general interest with regard to the construct validity is whether the structure, content and measurement results of a measurement procedure are consistent with existing theories (convergent validity). The descriptions of the severity of AB are normally a single factor [11-15, 19-25]. Characteristically, “the” intensity or “the” severity of AB is recorded. These single factor assumptions imply that changes in the intensity of the AB symptoms proceed in parallel (monotonic relationship). This means for example, that if the intensity of one symptom declines it must also decline for the others.

Two more specific questions are paramount for the BSS-ped: 1) Do the items reflect “the” severity of AB in children? The specific assumption is that the items substantially correlate in intra-individual comparison. 2) Do measurement results show changes? The studies, in which observation periods that started at the latest 48 hours after the first signs were registered, indicate a continuous decline of symptoms associated with coughing. This is confirmed by the results derived from placebo groups in placebo-controlled trials with observation periods of eight days [11, 12, 14, 15, 19, 20, 24, 26], seven to nine days [23] and eleven days [27, 28]. In line with previous findings, statistically significant decreases in measurement values should therefore occur during the course of the investigation. The accuracy of both construct validity criteria was to be investigated in the main and comparative samples.

#### 2.2.3 Criterion Validity and Testing Options

Criterion validity concerns the empirical relationship of a measurement result recorded by a measurement instrument - the BSS-ped in this case with a target criterion that is more (convergent) or less (discriminant) related to the construct. Criterion validity is sub-divided into the concurrent validity and predictive validity.

Concurrent validity corresponds to the correlation between two variables. Data were available for the following variables: FGK, Coughing Fits and Waking up at Night, KINDL Standard Questionnaire, KINDL Additional Subscale ‘Disease’, IMPSS and IMOS. They are presented subsequently.

##### 2.2.3.1 FGK

Using the FGK (Fragebogen zum Gesundheitszustand für Kinder [29] Child Health Questionnaire). The current state of health and quality of life of the patients was assessed according to the six items of the FGK (It’s all too much for me; I feel ill; I’m frightened; Playing or learning is difficult; I sleep badly; I find it hard to talk to others) each with five ratings between “not at all” to “definitely”. In children from the age of 1 to 6 years, the daily FGK assessment was carried out by the parents of the children. A factor analysis [30, 31] of the findings at the first visit, where all patients were untreated, showed that the items form a general factor that was not affected by age or sex. An FGK total score could therefore be determined by the summation of the item scores.

##### 2.2.3.2 Coughing Fits and Waking up at Night

The frequencies of coughing fits and waking up at night over the past 24 hours were to be documented in a carer’s diary.

##### 2.2.3.3 Long-term Health-related Quality of Life (KINDL)

The KINDL [31, 32] questionnaire used requires the carer of the patient, usually the mother, to assess the health-related quality of life on days 0 and 7 on the basis of 52 items, and to characterize the sick child over and above the acute condition. The details per item range from 1 = never, to 2 = seldom, through to 5 = all the time. Example: “My child was seldom tired and worn-out” or “My child felt strong and full of energy all the time”.

*KINDL Standard Questionnaire.* The first 24 items of the KINDL form one entity, divided into six subscales (physical well-being; emotional well-being; self-esteem; family; social contacts and kindergarten/school) that can be used to determine a total score (KINDL-total), since they loaded on a general factor in factor-analyses for visits 1 and 3. The KINDL questionnaire covers the time period of the last week.

*KINDL Additional Subscale ‘Disease’ and Kiddy-KINDL Parent.* Six further items, the so-called “KINDL Additional Subscale Disease”, refer to the periods when the patient was in hospital or was or is still suffering from a prolonged illness (item examples: I was afraid that my illness might get worse, I was sad because of my illness. Possible answers: 1 = never; 2 = sometimes; 3 = very often). These variables do not characterize the construct “acute bronchitis” and moreover do not hold for the condition of many patients. The same applies to the additional 22 items (possible answers: 1 = never; 2 = seldom; 3 = sometimes; 4 = often; 5 = all the time) that form the subscale “Kiddy-KINDL Parents”. The quality of life of the child, as the parents frequently experience it, was to be monitored by this means. Item examples of this subscale are: My child was moody and whined a lot; I had to give my child a telling-off; My child succeeded at everything he set out to do.

##### 2.2.3.4 Consistency with Treatment Satisfaction (Integrative Medicine Patient Satisfaction Scale; IMPSS)

Treatment satisfaction should reflect the change in AB severity. On the third visit (day 7), the extent of the patient’s satisfaction with treatment was assessed by the patient-carer using the IMPSS (Integrative Medicine Patient Satisfaction Scale) [33] according to the rating scale: 1 = very satisfied; 2 = satisfied; 3 = neutral; 4 = dissatisfied; 5 = very dissatisfied.

##### 2.2.3.5 Consistency with Treatment Success (Integrative Medicine Outcomes Scale; IMOS)

In the case of treatment, the extent of improvement or deterioration of AB should be related to the estimated success or failure of this treatment. The IMOS (Integrative Medicine Outcomes Scale) [33] is a single scale, where the investigator and the patient-carer are asked to independently assess the degree of treatment success in five categories: 1 = complete recovery; 2 = major improvement; 3 = slight to moderate improvement; 4 = no change; 5 = deterioration.

#### 2.2.4 Specific Hypotheses

If two variables are to measure the same construct, such as the acute status of health-related quality of life and the satisfaction with treatment and treatment success, a substantial correlation is to be expected (convergent validity). Thus, the BSS-ped should substantially correlate with the FGK total score and the Coughing Fits and Waking up at Night at all three visits [27, 28]. For IMPSS and IMOS positive correlations are also to be expected. Acute conditions of health-related quality of life as well as satisfaction and success of treatment can be expected to show a substantial negative correlation (convergent validity). With different constructs, such as *e.g*. long-term quality of life, the correlation should amount to a value around 0 (discriminant validity). This should also be true for age and sex, as was already mentioned in section “Description of Samples”. On the contrary, negative correlations are to be expected for the KINDL standard version, because, as a disease, AB is diametrically opposite to a healthy state [31, 32]. The symptoms are felt to be unpleasant and accordingly reduce the quality of life. With operationalisation through measurement instruments, it can be expected that the BSS-ped total scores 1) substantially correlate with measurement scores of acute state of health and quality of life and 2) act as indicators of the reduced acute state of health during the course of AB.

In addition, the BSS-ped differences between visits 1 and 2 should be less than between visits 1 and 3. Consequently, it is also to be expected that the correlations of the values of the differences with the IMOS score between visits 1 and 2 are not as high as between 1 and 3.

Concerning the average success of treatment of the placebo patients, the IMOS scores increase from the second to the third visit as assessed by relatives and investigators (3.00 -> 2.62). The changes also occur for the patients treated with an active substance.

From a validity point of view, it can be expected that marked correlations may be determined. However, after approximately one week this association is found [34, 35]. This may be due to the fact that the health-related quality of life, which is actually assessed as being “stable”, in fact declines under the influence of AB.

The predictive validity (prognostic validity) refers to the degree of probability with which future events can be predicted. Referring to the predictive validity, in particular, a significant decrease in BSS-ped value is expected in patients treated with placebo during the 8-day observation period [11, 12, 14, 15, 19, 20, 24, 26]. Furthermore, this will be slower than in patients who have been treated with a proven active substance [11-15].

#### 2.2.5 Overall Assessment of Validity

From the empirically-determined answers to these questions, a general conclusion may then be drawn as to whether the BSS-ped is adequately valid for common use in children. The germane question is: Can the validity of the BSS-ped be assessed as satisfactory according to the different aspects to be investigated here?

#### 2.2.6 Statistical Procedures

All analyses were performed with SPSS 17. For the presentation of quantitative variables, the following descriptive statistics were used: mean, median, standard deviation, minimum and maximum. Missing data were replaced by the last available value for each patient according to the LOCF method (last observation carried forward).

Nonparametric statistical procedures were preferentially used for determining correlations and performing significance tests. Principal component analysis according to Hotelling [36] with varimax-rotations according to the Kaiser criterion [37] was performed at a parametric level because this is adequate for this statistic computed by SPSS 17. Owing to the small sample sizes, frequently comprising of 39 or fewer persons, we accepted correlations as substantial if they differed from the null correlation at the 5 percent level.

#### 2.2.7 Ethics Committees

The data used for this analysis originated from investigations that had been approved by ethics committees [11, 15].

## 3 VALIDATION AND RESULTS

### 3.1 Content Validity

The BSS-ped is based on the specialised knowledge of pulmonology experts, who have a demonstrable presence within the scientific community through their scientific writings, amongst others, and for this reason, have an established reputation. According to Google Scholar [38], the publications of clinical trials in which the BSS was used, with first authors Matthys [14, 20, 21], Kamin [15], Chuchalin [19], Cwientzek [22], Gruenwald [23] and Haidvogl [24], are quoted in international scientific literature between 21 and 139 times in individual cases and around 60 times on average. The substantive considerations about the suitability of the BSS-ped items and about the demands put on this scale for measuring the severity of AB in children are therefore cogent.

Reliance on the academic standing of the pulmonology experts who deal with and use the BSS-ped or the BSS renders an additional literature search for other symptoms that are associated with AB and its severity in children superfluous. It should only be mentioned that the BSS-ped also includes the most frequently given symptom in this syndrome, *i.e*. coughing [29, 39-42], which is assessed by medical investigators as a matter of course.

Furthermore, the transfer of expertise into practical applications for using the BSS-ped in hospitals and medical practice will not reduce its validity, as the use of the scale requires no special training. After all, recording and rating of the items included in the scale pertain to medical training and medical routine.

### 3.2 Construct Validity

Although content validity has already clarified that the items cover the construct “acute bronchitis” (section “Content Validity”), their interrelationship remains an open question. As regards validity, there is no assumption about how they relate to each other in cross-sectional (inter-individual) comparisons. For the sake of completeness, this is also to be ascertained below, because a measurement procedure that “entirely” covers the construct is occasionally required for construct validity. However, the validity criterion requires that the comparative changes in severity move in the same direction (in parallel).

#### 3.2.1 Description of Samples

How the items are interrelated in inter-individual comparison can be investigated for the main and comparative sample with the full samples at the first visit, since at this point in time no experimental conditions (placebo, active substance preparations) have affected the patients.

The age of the children in the main sample was 2.82 + 0.53 (mean + standard deviation) years, ranging between 1 and 6 years. The statistics of the symptoms with the severity between 0 = absent and 4 = very severe and of the BSS-ped total score are given in Table **1**. This is also shown for the patients of the comparative sample, who were 3.76 + 1.59 years old and had an age range of between 1 and 6 years.

The correlations between the three items of the BSS-ped in the main sample lie between r = -0.12 and + 0.15 and do not differ significantly from null. In the comparative sample, they amount to r = -0.30 to + 0.22 and reach the level of significance in two cases. Following conversion to coefficients of determination (-0.30)^2^ = 0.09, *i.e*. 9% common variance), the highest correlation also proved to be non-relevant. It can, therefore, be assumed that the items are independent in the cross-sectional comparison and together cover a relatively large range of AB symptoms, *i.e*. they are not redundant.

Age and sex in both samples essentially show no correlation with the symptoms or the BSS-ped total score and therefore do not influence the measurement results.

#### 3.2.2 Severity of Acute Bronchitis

It is not the diagnosis of AB that is of primary interest from the validity point of view but the severity of the syndrome.

With five different degrees of intensity for each of the three items, *i.e*. a total of 13 possibilities of severity discrimination, the prerequisites for satisfactory validity of the BSS-ped are favourable. The distinct intensities, with five different degrees of severity to be evaluated per item, are in the optimum range for assessment differentiation.

As discussed above, the severity of the three symptoms has been shown to be independent in cross-sectional investigations at the first visit in both the main sample and the comparative sample.

Half the patients of not only the main sample but also the comparative sample were placebo patients (Table **2**). The age in the main sample was: 3.59 + 1.82 (m + sd) years and ranged between 1 and 6 years. 14 of the patients were female (35.9%). The corresponding statistics in the comparative sample are 3.77 + 1.77 years, between 1 and 6 years old, 18 patients were female (51.4%).

The changes during the course of the AB correlate well in the placebo patients but the age and sex of the patients were of no relevance (Table **3**). For each of the two samples with placebo patients, a structural analysis by means of principal component analysis was performed. The results, which are not given in detail here, clearly show a general factor for the change of severity, on which only the three symptoms load (highly), but not age or sex. This confirms that the BSS-ped is suitable for one-dimensional measurement of disease change and justifies the application of this short scale as a measure of the severity of AB in children. These findings were confirmed for the active substance patients (not given in detail here).

#### 3.2.3 Decline of Acute Bronchitis over the Observation Period

As comparisons of the occurrence of symptom intensity (Table **2**) and of the syndrome (Table **4**) show, the severity in the main and comparative samples decreased from visit to visit as expected. Significance tests were performed by means of the Friedman test. The results are only presented for placebo patients because they do not depend on the efficacy of an active substance (Table **5**).

The cross-validation with the patients receiving active treatment essentially led to the same results (without statistical evidence here).

Overall, the validity criterion, according to which the symptoms of AB decline over the observation period, is met and a satisfactory sensitivity to change can be assumed.

### 3.3 Criterion Validity

As the main sample partly encompassed other target criteria than the comparative study and as the items of the latter study were also in a different item context, *i.e*. measured within the total BSS, only the main sample will be considered below.

#### 3.3.1 Conformity Validity

Hypotheses on the results of the variables taken with BSS-ped explicated under 2.2.5 “Specific hypotheses” are affirmed with the exception of r (BSS-ped with FGK) at the visits 1 and 3 (Table **6**): FGK total scores, the Coughing Fit and Waking up at Night, the IMPSS and IMOS displayed positive and the KINDL standard version negative correlations to BSS-ped total scores (Table **6**). Besides, the means of BSS-ped total scores, FGK scores and numbers of Coughing Fits and Waking up at Night declined significantly.

##### 3.3.1.1 Health and Quality of Life

The courses of the means of the FGK and BSS-ped behaved as expected: They declined consistently in both scales from visit to visit (Table **6**).

##### 3.3.1.2 Coughing Fits and Waking up at Night

The frequencies of coughing fits and waking up at night over the past 24 hours were to be documented in a carer’s diary. As both events are associated with the severity of AB [27, 28], they should also correlate with the BSS-ped score as follows:

1. The higher the patient’s BSS-ped total score is, the higher the patient’s number of coughing fits or frequency of waking up at night is likely to be. A close relationship with the BSS-ped total score is not to be expected, however, because the frequency of coughing fits and waking up at night is probably not very reliable, as they are recorded by only one daily question and their occurrence may also be influenced by other factors – being woken up, for example, due to the evening meal or due to light and noises in the surroundings, and on whether the event is actually noticed by the family member looking after the child,* etc*.
2. With the decrease in severity of AB the frequency of coughing fits and waking up at nights should also decline. Both correlate moderately, even after controlling for the influence of age and sex (first visit: r_coughing fits-waking.age-sex_ = 0.377; *p* = 0.001; 1-tailed test). The correlations between coughing fits and waking up at night and the BSS-ped determined per visit range between “not” and “highly significant” (Table **6**) and only partially fulfil the 1^st^ assumption.

**Fig. (1):**
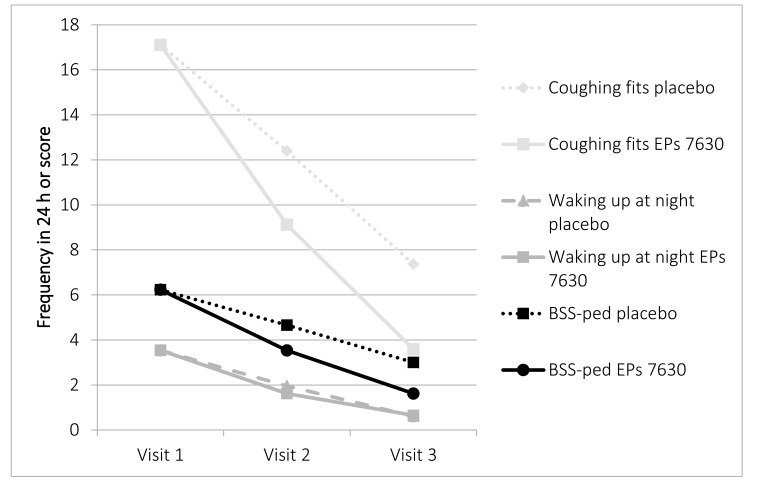
Mean course of coughing fits and waking up at night as well as the BSS-ped total scores in the patients receiving placebo (n=39) and active substance (EPs 7630; n=39), respectively, of the main sample (at Visit 1 still untreated; therefore the two groups are not yet split).

On the other hand, the means of the BSS-ped and of the coughing fits and frequency of waking up at night each continuously decrease from visit 1 to 3 as expected, which is completely consistent with the 2^nd^ assumption (Fig. **1**).

The so-called “KINDL Additional Subscale Disease” refers to periods when the patient was in the hospital or was or is still suffering from a prolonged illness. No substantial relationship with the BSS-ped score was expected for either scale. In the sense of discriminant validity, this assumption was confirmed (Table **6**).

As expected, the extent of the BSS-ped score difference between visits 1 and 3 correlated with treatment satisfaction in the placebo patients (Table **6**).

In addition, the correlations of the values of the differences with the IMOS score between visits 1 and 2 are not as high as between 1 and 3, but are still significant.

Fig. (**2**) shows that the BSS-ped scores decreased from visit to visit for both the placebo and the active substance patients. As expected, the average success of treatment of the placebo patients increased from the second to the third visit as assessed by relatives (3.10 -> 2.72) and investigators (3.00 -> 2.62). The changes for the patients treated with active substance amounted to 2.69 -> 1.85 (relatives) and 2.59 -> 1.85 (investigators). The previously (cf. to chapt. 2.2.5) stated assumption is correct.

Without exception, the same applies for the correlations between the extent of the differences in the BSS-ped scores and the assessments of the treatment success in the placebo and active substance patients by the investigators and the patient carers (Table **6**).

#### 3.3.2 Prognostic Validity

After partialling out the influence of age and sex, the BSS-ped total score for the placebo patients correlated with the first Visit as shown in the following visit 2: r = 0.73 (*p* = 0.000), Visit 3: r = 0.50 (*p* = 0.001). This result confirms one of the two assumptions about prognostic validity.

In order to test the other prognosis, the placebo and active substance samples were compared. It is assumed that the active substance specifically administered, in this case the *Pelargonium sidoides* preparation EPs 7630, is in fact effective. Its action has been confirmed in many studies [11-15, 19-21, 24, 26, 43-45]. Therefore, it can be taken that the assumption about efficacy is true.

Without treatment, *i.e*. at Visit 1, there was practically no difference between the BSS-ped scores of the placebo and active substance groups (*p* = 0.391 - Mann Whitney-U-Test, exact, 1-tailed test). The BSS-ped scores of the active substance patients were significantly lower than those of the placebo patients at Visit 2 (*p* = 0.000) and Visit 3 (*p* = 0.000).

The assumptions of predictive validity are therefore confirmed for the BSS-ped total scale.

## 4 DISCUSSION

The BSS-ped was tested according to classical measurement and test theory in all important aspects with regard to whether it is valid in terms of being able to objectify the severity of AB in children.

### 4.1 Content Validity

Whether the scale fits into the framework of state of the art pneumology with regard to content could not be tested directly on the basis of existing theories about AB, its severity and measurement, but only indirectly, as this area is still evolving. The corresponding research therefore depends on the pioneers in this area. For external parties, the qualitative level of scientific work can be most reliably assessed by recognition in the scientific community [18, 46]. The impact of the scientists working in the field of the BSS-ped through publications and the acceptance of these publications, as reflected by citations in international scientifically renowned journals and book contributions, demonstrates that the qualitatively high level of their work is fundamentally recognized by the scientific community. Equally, it cannot seriously be doubted that the BSS-ped has at least superior content quality based on the research findings of the pneumologists and pediatricians of high standing in their professional circles. The level of BSS-ped content validity may therefore be evaluated as high.

### 4.2 Construct Validity

The following three characteristics constitute the key elements of the AB severity construct:

1. AB comprises at least three central symptoms, *i.e*. coughing, pulmonary rales at auscultation, and dyspnoea;
2. The severity of which changes in parallel (monotonic relationship);
3. The duration of the disease may extend for up to several weeks.

The tests of the BSS-ped show that it meets the validity requirements with regard to these three key elements of AB severity in the two patient samples investigated.

For the first of the key elements, it should be noted that the three central symptoms are not correlated with each other in inter-individual measurements and, therefore, reflect a relatively broad spectrum of symptoms partially evolving sequentially, which is desirable in terms of avoiding redundancy. However, the severity of these symptoms changes in parallel. The exception, which concerns the item dyspnoea, appears to be a methodological artifact, since the median at the second and the third visit amounted to = 0.0 and the variance of its score was greatly reduced. It can therefore be assumed that the BSS-ped total score reflects one-dimensional severity of AB.

In spite of the satisfactory results, the correlations of the changes found are still an underestimate of the actual situation, since the study enrolment criterion for the patients with acute bronchitis was at least five total points in the standard version of the five-item BSS (*e.g* [11, 14, 15, 34]). This selection resulted in a homogeneous sample compared to non-selected samples. Owing to the larger range of AB severity, higher correlations between the results of temporally contiguous measurements were to be expected.

### 4.3 Criterion Validity

Criterion validity was investigated in the patients receiving placebo with regard to the correlative relationship between measurement results in the BSS-ped and a validity criterion for a total of 18 criteria (Table **6**). For twelve criteria, an additional cross-validation was performed in active substance patients (Table **6**), because there was no evidence that the effect of the active substance influenced the respective validity aspect of interest.

There was a more or less close relationship to the construct of AB severity for the criteria. For nine criteria (Table **6**) closer empirical links with AB measurement results were expected, for six moderate but still statistically significant relationships and for three no convergence (divergent criteria).

All the criteria associated with the construct correlated significantly with the BSS-ped results (Table **6**). This implies high correlations also among these clinically particularly relevant criteria. This was checked for the IMOS as rated by physicians and patients´ legal representatives, as well as for the IMPSS of treated and untreated patients (n=38 instead of 39 in each treatment group because of 1 noncompleted data set) (Table **7**). There was a high correlation between the treatment success ratings (IMOS) performed by physicians and legal representatives (r=0.879). Also for the evaluation of patient satisfaction (IMPSS) a relatively high correlation with the treatment success ratings could be shown (r(IMPSS-IMOS_physician_) = 0.603; r(IMPSS-IMOS_legal representatives_) = 0.690).

In one case, which relates to the FGK at the first visit, the correlation was quite low, however, with 0.28. For the interpretation, particularly at the first visit, the fact that the “true” relationship is underestimated should be taken into account, because very mild and mild AB, as mentioned above, were not included. The samples were therefore homogenous with regard to severity.

Furthermore, the criteria such as IMOS and IMPSS comprised only one item. Several other scales, such as the FGK and KINDL supplementary questions, whose measurement results also served as validity criteria, comprised only six items. For this reason, their reliability was already limited. This reduced the ascertainable validity coefficients, which, apart from the given exception, were between 0.41 and 0.76 for the validation and cross-validation and therefore at a satisfactory level.

Moderate relationships to the number of coughing fits and waking up at night during the previous 24h were expected. These criteria comprise only one item each and are dependent on many conditions. For this reason, the three low but nevertheless significant correlations compared to three non-significant correlations (Table **6**) may still be interpreted as proof of validity. In the cross-validation, there is one significant correlation compared to four non-significant correlations.

At the first visit, no relationship with the measurement values in the three KINDL scales was expected, because they referred to long-term or prevalent characteristics and not to the acute status. These assumptions were found to apply (Table **6**) and thus also confirm validity.

The assumptions made from the criterion validity viewpoint of parallel decline during the course of the disease for the BSS-ped and FGK scores as well as the number of coughing fits and waking up at night – not included in Table **6** were confirmed without exception (in part portrayed in Fig. (**1**)).

On top of this, each of the four assumptions tested for prognostic validity satisfied the requirements to a highly significant degree.

### 4.4 Confounder

The present report has repeatedly demonstrated that the BSS-ped scores are not systematically influenced by age or sex in the one to six-year-olds. This result may also be interpreted as further proof of validity under the aspect of divergent validity.

The data collected were mainly based on assessments. Here, the influence of auto- and hetero-suggestion as confounders, which lead to intentional or unintentional distortions in the expected results, should always be taken into consideration [47]. For example, improvements in the state of health of placebo patients could result from them being influenced by the suggestion that they were being effectively treated. The younger the child, the less likely he/she is liable to be influenced by such suggestions. There was thus no relationship with age within the one to six-year-old patient group.

Assessments by the investigators and patient’s relatives are more likely to be subjected to the hetero-suggestion that AB improves with treatment. This cannot be completely excluded in the assessment of treatment success and satisfaction, but does not explain the superiority of active treatment over placebo because the conditions were double-blind. Furthermore, there was no evidence that the validity aspects investigated here were affected.

Suggestive influences on data collection may be excluded for several reasons: The data used for the validity analyses were collected for completely different reasons than for validation purposes. On top of this, other people were involved in actual data collection than the persons who developed the scale. The transfer to electronic data media was undertaken by other persons again, none of whom were concerned with the validation.

The original pursuit of other objectives than validation and the distribution of tasks during the acquisition and analysis of data, on which the validity testing is based, thus render the results to be particularly reliable.

### 4.5 Overall Assessment of Validity

The findings regarding content validity, validity of the scale structure (construct validity) and criterion validity, which have been confirmed to a greater or lesser degree by cross-validation, show the BSS-ped to be a valid and sensitive measuring instrument for recording the severity and change in severity of AB in children.

Indeed, validity testing of the scale structure was carried out using the course changes of AB in 78 patients, *i.e*. a not a very large sample. Cross-validation with an independent sample of a further 70 patients between one and six years of age with the same diagnosis confirmed the validity findings. It is therefore not to be expected that further validation studies with additional or larger samples will lead to other results.

The same applies to criterion validity testing, which was performed with 39 placebo patients and cross-validated with a similar sized active substance group.

### 4.6 Scope of Application and Generalisability

The use of the BSS-ped is probably not restricted to one to six-year-old children but can also be extended to younger as well as older children and to adolescents. This is suggested by the results of the five-item BSS, which was recorded in patients up to 92 years of age. As the BSS data analyses show, the three items chosen for the BSS-ped load on the same factor, which was determined by principal component analyses. It is described there as the “coughing complex” [34, 45]. The BSS-ped is independent of the second factor (“sputum”). It measures the severity of AB associated on the whole with the coughing complex in pre-school as well as school children and adolescents.

The one-dimensionality of AB severity measured by the BSS-ped applies, strictly speaking, for the period of eight days following the doctor’s visit, which in turn takes place within 48 hours of initial perception of the child’s illness by those responsible for the patient. Whether the symptom severity and the correlation of the changes in the period subsequent to the eight days following the first visit still behave in the same manner cannot be empirically investigated from the available data.

The three symptoms of the BSS-ped in connection with AB are widely disseminated in the scientific community, as shown by internationally available publications [11, 14, 15, 19-24]. For this reason, the physician’s symptom assessment, as required by the BSS-ped, should not present any problems across language barriers. Furthermore, the application of the BSS-ped in medical circles worldwide should be possible without any systematic loss of validity.

The individual characteristics of the investigators also appear to have no critical impact on the rating. This is also reflected in the sometimes high correlations between the BSS-ped and the validity criteria found here, based on the assessments of 21 different physicians in two countries. If the ratings of the scale were not very objective, *i.e*. not inter-subjectively largely identical, then such high correlations with the validity criteria would not be found.

### 4.7 Practicability of Rating

The BSS-ped is very practical. It can be recorded by persons with medical competence in patients during visits without the verbal assistance of the latter and without specific technical equipment. It is sensitive to syndrome changes and displays them at time intervals of a few days, probably over even shorter periods.

## CONCLUSION

The BSS-ped has been shown to be a short scale sensitive to change that is independent of the age and sex of the assessed one to six-year-old patients with AB. It records the one-dimensional severity of this syndrome. Using this scale, the investigator can assess the severity of the cardinal symptom coughing and the two other symptoms of importance in AB: pulmonary rales at auscultation, and dyspnoea. The scope appears to encompass all medical applications that are geared towards the international scientific community. Within this framework, there are in principle no national boundaries for its use.

The possibilities of medical severity assessment per BSS-ped item are already standardized to “0 = absent”, “1 = mild”* etc*. to “4 = very severe”. Therefore, it seems reasonable to transfer this graduation to an overall scale as depicted in Table **8** with a slight interpolation (Interpretation: “unclear”). Through this standardization in the interpretation of the measurement results, the BSS-ped advances from “just” a measurement instrument to a test, *i.e*. to a qualitatively higher level [16, 17]. It can thus be used as a clinical test procedure in medical practice and research for standardised objectivation of the severity of AB in children, assuming that the diagnosis “acute bronchitis” has already been made or is under consideration.

Fig. (**2**) shows a graphic presentation based on mean courses of illness in the placebo and active substance patients of the main study.

The qualities objectivity and reliability, that are often considered separately, were not individually measured, as these are implied in validity according to the following rule: The validity cannot be lower than the reliability and the latter not lower than the objectivity [16, 17]. On the other hand, high objectivity and reliability are requirements for high validity only, but do not guarantee this. Finally, the degree of validity and not the extent of objectivity or reliability is the measure for the “correctness” of the assertions that can be made from the measurement results.

## Figures and Tables

**Fig. (2):**
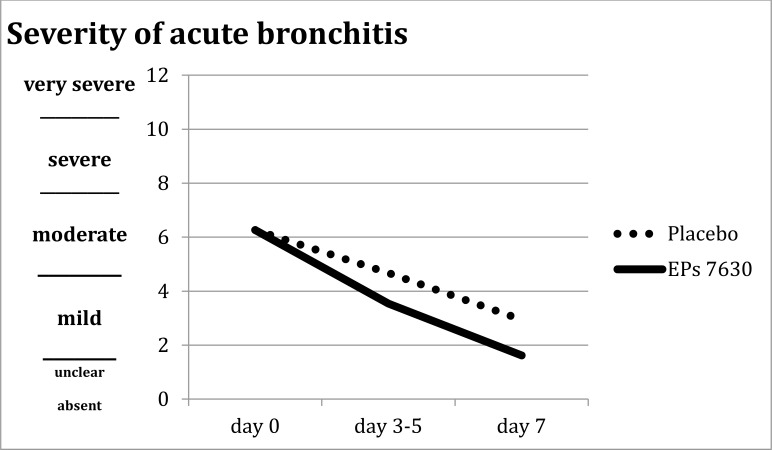
Course of severity (arithmetic means of the BSS-ped scores) of acute bronchitis in one to six-year-old patients in the main sample (n=39 each).

**Table 1:** Basic statistics for the three items of the BSS-ped and of the BSS-ped total score of the 1-6-year-old patients in the main and comparative samples at the first visit.

-	Main Sample: n=78				Comparative Sample: n=70			
-	Items of the BSS-ped			total	Items of the BSS-ped			total
-	Cough	Rales°	Dyspnoea	BSS-ped	Cough	Rales°	Dyspnoea	BSS-ped
Arithmetic Mean m	2.82	2.36	1.08	6.26	2.39	1.91	0.39	4.69
Median md	3.00	2.00	1.00	6.00	2.00	2.00	0.00	5.00
Standard Deviation sd	0.53	0.66	0.80	1.18	0.57	0.50	0.55	0.91
Minimum	2	1	0	5	1	1	0	3
Maximum	4	4	3	10	4	3	2	7

°pulmonary rales at auscultation

**Table 2:** Percentage incidence of estimated severity per item of the BSS-ped at the three visits of the placebo patients.

			Main Sample: n = 39		Comparative Sample: n = 35*		
Visit	Severity	Cough	Rales	Dyspnoea	Cough	Rales	Dyspnoea
1	Absent	0.0	0.0	28.2	0.0	0.0	60.0
	Mild	0.0	7.7	46.2	2.9	14.3	34.3
	Moderate	23.1	46.2	23.1	65.7	77.1	5.7
	Severe	66.7	46.2	2.6	28.6	8.6	0.0
	Very severe	10.3	0.0	0.0	2.9	0.0	0.0
2	Absent	0.0	2.6	71.8	0.0	0.0	74.3
	Mild	5.1	23.1	25.6	2.9	20.0	22.9
	Moderate	56.4	43.6	2.6	74.3	68.6	2.9
	Severe	38.5	30.8	0.0	20.0	11.4	0.0
	Very severe	0.0	0.0	0.0	2.9	0.0	0.0
3	Absent	2.6	23.1	94.9	2.9	11.4	77.1
	Mild	30.8	41.0	5.1	22.9	37.1	14.3
	Moderate	59.0	30.8	0.0	57.1	37.1	8.6
	Severe	7.7	5.1	0.0	14.3	14.3	0.0
	Very severe	0.0	0.0	0.0	2.9	0.0	0.0

*Taken from the BSS

**Table 3:** Partial correlation of the BSS-ped items and the total score between consecutive visits of the placebo patients in the main and comparative sample after partialling out age and sex.

-	Main sample: n = 39		Comparative sample: n = 35*	
Variable	Visit 1 with Visit 2 (p-value)**	Visit 2 with Visit 3 (p-value)**	Visit 1 with Visit 2 (p-value)**	Visit 2 with Visit 3 (p value)**
Cough	0.33 (0.002)	0.54 (0.000)	0.41 (0.009)	0.74 (0.000)
Rales°	0.60 (0.000)	0.66 (0.000)	0.38 (0.015)	0.64 (0.000)
Dyspnoea	0.56 (0.000)	0.51 (0.000)	0.68 (0.000)	0.79 (0.000)
BSS-ped	0.55 (0.000)	0.72 (0.000)	0.43 (0.006)	0.82 (0.000)

* Taken from the BSS; **significance-test 1-sided; **°** pulmonary rales at auscultation

**Table 4:** Proportional distribution of BSS-ped total score at the first, second and third visit of the placebo patients.

-	Main Sample: n = 39			Comparative Sample: n = 35*		
Severity (Total score)	Visit 1 Day 0	Visit 2 Day 3-5	Visit 3 Day 7	Visit 1 Day 0	Visit 2 Day 3-5	Visit 3 Day 7
0	0.0	0.0	2.6	0.0	0.0	2.9
1	0.0	2.6	17.9	0.0	0.0	5.7
2	0.0	2.6	15.4	0.0	2.9	20.0
3	0.0	7.7	28.2	8.6	14.3	17.1
4	0.0	33.3	23.1	37.1	42.9	22.9
5	25.6	28.2	7.7	34.3	25.7	17.1
6	41.0	20.5	5.1	14.3	11.4	2.9
7	20.5	2.6	0.0	5.7	0.0	8.6
8	10.3	2.6	0.0	0.0	0.0	0.0
9	0.0	0.0	0.0	0.0	2.9	2.9
10	2.6	0.0	0.0	0.0	0.0	0.0
11	0.0	0.0	0.0	0.0	0.0	0.0
12	0.0	0.0	0.0	0.0	0.0	0.0

* Taken from the BSS

**Table 5:** Results of significance tests regarding the course of change of the BSS-ped total and the item scores from the first to the third visit (mean ranks) in placebo patients of the main and comparative sample using the Friedman-Test.

-	Main sample: n = 39				Comparative sample: n = 35*			
-	Cough	Rales°	Dyspnoea	BSS-ped	Cough	Rales°	Dyspnoea	BSS-ped
Day 0	2.67	2.56	2.92	2.67	2.20	2.16	2.14	2.33
Day 3-5	2.01	2.17	2.00	2.01	2.09	2.14	1.90	2.06
Day 7	1.32	1.27	1.08	1.32	1.71	1.70	1.96	1.61
Chi^2^ (df = 2)	52.51	50.51	72.00	52.51	12.90	9.32	4.27	12.37
Exact significance (p)	0.000	0.000	0.000	0.000	0.001	0.009	0.115	0.002

* Taken from the BSS; °pulmonary rales at auscultation

**Table 6:** Correlations between the BSS-ped total and other scales that are close to the construct “acute bronchitis” to a greater (convergent) or lesser (discriminant) extent in the – if not stated otherwise – 39 placebo patients (in brackets: 39 active substance patients).

**Close to the Construct “Acute Bronchitis”**	**BSS-ped in Correlation with**	**Item**	**Partial ** **Correlation r**^ 0^	**Significance: *p* value (1-tailed)**
	**FGK, Visit 1**^1^	Acute state of health, acute quality of life	0.28	0.010
	**FGK, Visit 2**	Acute state of health, acute quality of life	0.45 (0.46)	0.003 (0.002)
	**FGK, Visit 3**	Acute state of health, acute quality of life	0.44 (0.66)	0.004 (0.000)
	**KINDL standard questions, Visit 3**^2^	Sum of the six sub-scale scores: Physical well-being, emotional well-being, social contacts, kindergarten/school, self-esteem and family	-0.61 (-0.69)	0.031^3^ 0.000**^4^**
**Change in the Construct “Severity of Acute Bronchitis” Close to the Criterion**	**IMPSS, Visit 1 vs. Visit 3**^2^	Satisfaction with treatment	0.46 (0.63)	0.002^5^ (0.000)^5^
	**IMOS, Visit 1 vs. Visit 2, patient relatives**	Success of treatment	0.41 (0.60)	0.006^6^ (0.000)^6^
	**IMOS, Visit 1 vs. Visit 3, patient relatives**	Success of treatment	0.66 (0.67)	0.000^7^ (0.000)^7^
	**IMOS, Visit 1 vs. Visit 2, investigator**	Success of treatment	0.51 (0.52)	0.001^6^ (0.000)^6^
	**IMOS, Visit 1 vs. Visit 3, investigator**	Success of treatment	0.76 (0.62)	0.000 (0.000)
**Moderate Relationship with the** **Construct “Acute Bronchitis”**	**Coughing fits, Visit 1**^1^	Number of coughing fits in the past 24 h	0.02	0.439
	**Coughing fits, Visit 2**	Number of coughing fits in the past 24 h	0.24 (0.17)	0.084 (0.173)
	**Coughing fits, Visit 3**	Number of coughing fits in the past 24 h	0.30 (0.80)	0.037 (0.000)
	**Waking up at night, Visit 1^1^**	Number of times waking up at night in the past 24 h	0.27	0.013
	**Waking up at night, Visit 2**	Number of times waking up at night in the past 24 h	0.35 (0.17)	0.019 (0.182)
	**Waking up at night, Visit 3**	Number of times waking up at night in the past 24 h	0.05 (0.10)	0.382 (0.290)
**No Relationship to the Construct “Acute Bronchitis”**	**KINDL standard questions, Visit 1**^2^	Sum of the six sub-scale scores: Physical well-being, emotional well-being, social contacts, kindergarten/school, self-esteem and family	-0.04	0.416^7^
	**KINDL additional questions disease, Visit 1**	Periods when the patient was in hospital or was or still is suffering from a longer illness	-0.16	0.211^8^
	**Kiddy-KINDL parents, Visit 1**	Quality of life of a child from parent’s viewpoint	0.09	0.338

^0^after partialling out age and sex; ^1^all still untreated, therefore no splitting into placebo and active substance treatment; ^2^r (zero order); ^3^n = 10 (only complete series of data); ^4^n = 11 (only complete series of data); ^5^n = 38 (only complete series of data); ^6^n = 35 (only complete series of data); ^7^n = 26 (only complete series of data); ^8^n = 28 (only complete series of data) FGK: Fragebogen zum Gesundheitszustand für Kinder IMOS: Integrative Medicine Outcomes Scale IMPSS: Integrative Medicine Patient Satisfaction Scale

**Table 7:** (Product-moment) intercorrelations of age and sex with the BSS-ped results and the results of the criterion variables IMOS and IMPSS for treated (top triangular matrix) and untreated patients (bottom triangular matrix) of the main sample.

-	Age	Sex	BSS-ped Diff(visit 3 minus visit 1)	IMOS by Physician	IMOS by Legal Representative	IMPSS
Age	-	-.050	.037	.083	.108	.163
Sex	.087	-	.233	.029	-.053	.112
BSS-ped diff(visit 3 minus visit 1)	.280	.023	**-**	.549***	.592***	.629***
IMOS by physician	-.016	.072	.691***	**-**	.879***	.603***
IMOS by legal representative	.031	-.153	.613***	.804***	**-**	.690***
IMPSS	.013	-.020	.457**	.542***	.567***	**-**

**significant at 1-%-level ***significant at 1-‰-level IMOS: Integrative Medicine Outcomes Scale IMPSS: Integrative Medicine Patient Satisfaction Scale

**Table 8:** An overall scale for the interpretation of the severity of acute bronchitis based on the BSS-ped total scores.

**BSS-ped Total Score**	**Interpretation**
0	Absent
1	Unclear
2-4	Mild
5-7	Moderate
8-10	Severe
11 or 12	Very severe

## LIST OF ABBREVIATIONS

AB: = Acute Bronchitis
BSS: = Bronchitis Severity Scale
m: = Arithmetic mean
FGK: = Fragebogen zum Gesundheitszustand für Kinder
IMOS: = Integrative Medicine Outcomes Scale
IMPSS: = Integrative Medicine Patient Satisfaction Scale
md: = Median
LOCF: = Last Observation Carried Forward
SD: = Standard deviation
